# Ginsenoside Rb1, Compound K and 20(S)-Protopanaxadiol Attenuate High-Fat Diet-Induced Hyperlipidemia in Rats via Modulation of Gut Microbiota and Bile Acid Metabolism

**DOI:** 10.3390/molecules29051108

**Published:** 2024-03-01

**Authors:** Kang-Xi Zhang, Yue Zhu, Shu-Xia Song, Qing-Yun Bu, Xiao-Yan You, Hong Zou, Guo-Ping Zhao

**Affiliations:** 1 Henan Engineering Research Center of Food Microbiology, College of Food and Bioengineering, Henan University of Science and Technology, Luoyang 471023, China; zhangkx@tib.cas.cn (K.-X.Z.); asongshuxia@163.com (S.-X.S.); buqy@tib.cas.cn (Q.-Y.B.); 2 Master Lab for Innovative Application of Nature Products, National Center of Technology Innovation for Synthetic Biology, Tianjin Institute of Industrial Biotechnology, Chinese Academy of Sciences, Tianjin 300308, China; zhuyue@tib.cas.cn; 3 Haihe Laboratory of Synthetic Biology, Tianjin 300308, China; 4 CAS Engineering Laboratory for Nutrition, Shanghai Institute of Nutrition and Health, Chinese Academy of Sciences, Shanghai 200031, China; 5 CAS-Key Laboratory of Synthetic Biology, CAS Center for Excellence in Molecular Plant Sciences, Shanghai Institute of Plant Physiology and Ecology, Chinese Academy of Sciences, Shanghai 200032, China

**Keywords:** ginsenoside Rb1, ginsenoside CK, ginsenoside PPD, hyperlipidemia, gut microbiota, bile acid metabolism

## Abstract

Hyperlipidemia, characterized by elevated serum lipid concentrations resulting from lipid metabolism dysfunction, represents a prevalent global health concern. Ginsenoside Rb1, compound K (CK), and 20(S)-protopanaxadiol (PPD), bioactive constituents derived from Panax ginseng, have shown promise in mitigating lipid metabolism disorders. However, the comparative efficacy and underlying mechanisms of these compounds in hyperlipidemia prevention remain inadequately explored. This study investigates the impact of ginsenoside Rb1, CK, and PPD supplementation on hyperlipidemia in rats induced by a high-fat diet. Our findings demonstrate that ginsenoside Rb1 significantly decreased body weight and body weight gain, ameliorated hepatic steatosis, and improved dyslipidemia in HFD-fed rats, outperforming CK and PPD. Moreover, ginsenoside Rb1, CK, and PPD distinctly modified gut microbiota composition and function. Ginsenoside Rb1 increased the relative abundance of *Blautia* and *Eubacterium*, while PPD elevated *Akkermansia* levels. Both CK and PPD increased *Prevotella* and *Bacteroides*, whereas *Clostridium-sensu-stricto* and *Lactobacillus* were reduced following treatment with all three compounds. Notably, only ginsenoside Rb1 enhanced lipid metabolism by modulating the PPARγ/ACC/FAS signaling pathway and promoting fatty acid β-oxidation. Additionally, all three ginsenosides markedly improved bile acid enterohepatic circulation via the FXR/CYP7A1 pathway, reducing hepatic and serum total bile acids and modulating bile acid pool composition by decreasing primary/unconjugated bile acids (CA, CDCA, and β-MCA) and increasing conjugated bile acids (TCDCA, GCDCA, GDCA, and TUDCA), correlated with gut microbiota changes. In conclusion, our results suggest that ginsenoside Rb1, CK, and PPD supplementation offer promising prebiotic interventions for managing HFD-induced hyperlipidemia in rats, with ginsenoside Rb1 demonstrating superior efficacy.

## 1. Introduction

Hyperlipidemia is a critical factor in the onset of numerous diseases, including non-alcoholic fatty liver disease (NAFLD), obesity, and cardiovascular disease (CVD) [1]. Strategies to mitigate lipid metabolism dysfunction are pivotal in curbing the prevalence of hyperlipidemia [2]. The liver is central to systemic lipid regulation, overseeing processes such as cholesterol metabolism, fatty acid (FA) uptake and export, de novo lipogenesis, and β-oxidation [3]. Excessive cholesterol accumulation is detrimental, posing risks for various health conditions [4]. A primary pathway for cholesterol clearance involves its conversion into bile acids (BAs), which also function as signaling molecules and metabolic regulators, influencing hepatic lipid, glucose, and energy balance to maintain metabolic equilibrium [5,6]. The gut microbiota exerts a significant influence on cholesterol homeostasis and BA metabolism, transforming primary BAs into secondary BAs via several biochemical reactions [7]. Notably, the FXR/Fgf15 signaling pathway plays a key role in the reabsorption and secretion of intestinal BAs [8].

*Panax ginseng*, a perennial herb, is renowned for its diverse bioactive and medicinal properties, primarily attributed to its ginsenosides, polysaccharides, and peptides [9]. Ginsenosides, particularly the dammarane and oleanolic acid types, are the herb’s principal bioactive constituents [10]. Among these, ginsenoside Rb1 stands out for its potential to mitigate HFD-induced hepatic steatosis by modulating peroxisome proliferator-activated receptor γ (PPARγ) expression and intestinal microbial structure and function [11,12]. Compound K (CK) exhibits enhanced bioavailability and water solubility using nanocarriers and cyclodextrin for delivery, with its anti-diabetic and anti-hyperlipidemic effects the subjects of recent research [13,14]. Meanwhile, 20(S)-protopanaxadiol (PPD), a deglycosylated metabolite produced by gut microbiota, has demonstrated immunostimulatory effects [15]. However, the specific contributions of ginsenoside Rb1, CK, and PPD to hyperlipidemia progression in HFD-fed rats remain to be elucidated.

The interaction between ginsenosides and gut microbiota post-oral administration is crucial for human health [16]. The structural complexity of ginsenosides, including their polar surface area and the presence of sugar moieties, limits their direct pharmacological action [17]. The gut microbiota facilitates the transformation of ginsenosides into more bioavailable metabolites, such as CK and PPD, enhancing their pharmacological activity [18]. Despite existing research, comparative studies on the effects of ginsenoside Rb1, CK, and PPD on lipid metabolism in HFD-fed rats and the mechanisms involved are scarce. Our study aims to fill this gap by investigating the impact of these ginsenosides on hyperlipidemia and delving into the mechanisms related to gut microbiota composition and function, as well as the enterohepatic circulation of BAs. This research could unveil new natural product-based strategies for improving lipid metabolism disorders.

## 2. Results

### 2.1. Effects of Ginsenoside Rb1, CK, and PPD on Body Weight, Serum Biochemistry, Hepatic Adipose Accumulation in HFD-Fed Rats

**Figure 1:**
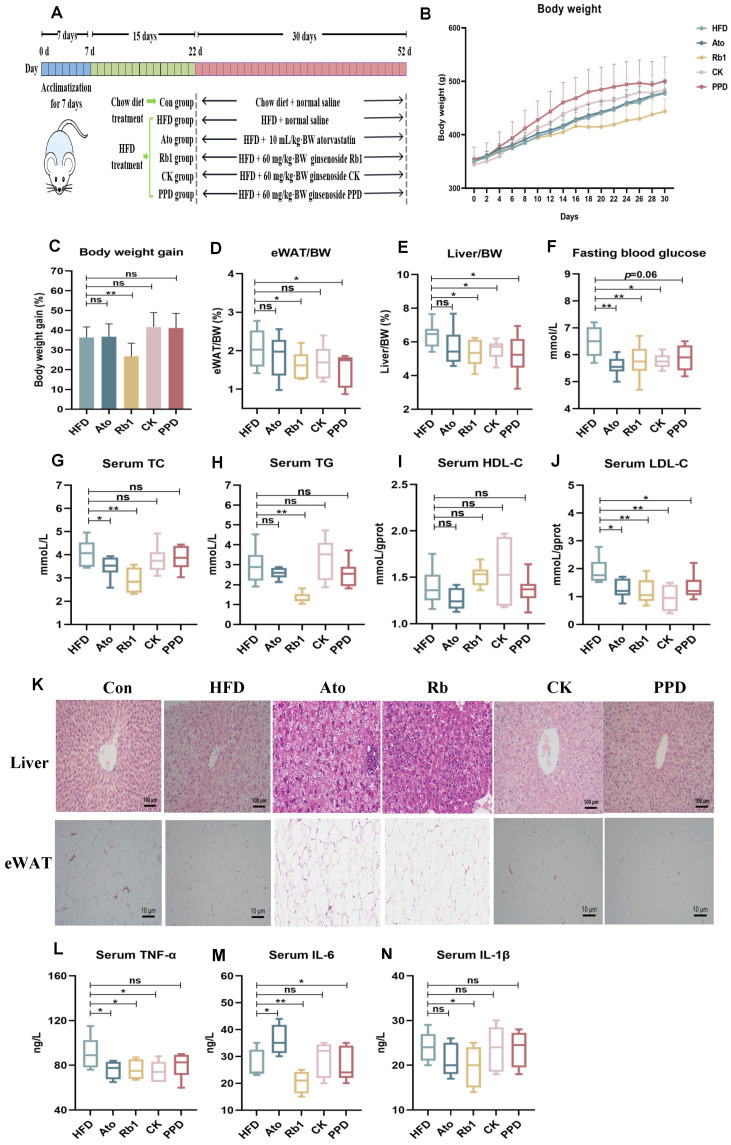
Effect of ginsenoside Rb1, CK and PPD treatment on body weight, hyperlipidemia and liver steatosis in HFD-fed rats. (**A**) The experimental design, (**B**) body weight (g), (**C**) body weight gain (%), (**D**) eWAT/BW (%), (**E**) liver/BW (%), (**F**) fasting blood glucose (mmol/L), (**G**–**J**) serum concentrations of TC, TG, HDL-C and LDL-C (mmol/L), (**K**) histological analysis for liver and epididymal adipose tissues (eWAT), and (**L**–**N**) serum concentrations of TNF-α, IL-6 and IL-1β (ng/L). Values are the means ± SEMs. * *p* < 0.05, ** *p* < 0.01 and versus the HFD group, ns means no significantly difference.

The animal experimental design was shown in Figure 1A. Compared with rats fed with standard diet (Con group), rats fed with a high-fat diet (HFD group) showed significantly increased BW and body weight gain (Supplementary Materials Figure S1A,B). Compared with the HFD group, Rb1 treatment dramatically reduced BW and body weight gain, while there was no significant difference in body weight gain among the PPD group, the CK group and the HFD group, indicating that the effect of ginsenoside Rb1 on improving weight gain was superior to that of ginsenoside CK and PPD treatment (Figure 1B,C). Further, eWAT/BW, liver/BW and fasting blood glucose (FBG) levels were decreased in the Rb1 group and the PPD group. Ginsenoside CK also reduced the eWAT/BW and FBG levels, whereas there was no significant difference in liver/BW between the CK group and the HFD group (Figure 1D–F). Furthermore, compared to the Con group, serum TC, TG, and LDL-C levels were higher and the serum HDL-C level was lower in the HFD group (Supplementary Materials Figure S1D). The Rb1 group showed significantly reduced serum TC, TG, and LDL-C levels compared to the HFD group. Although the serum LDL-C level was lower, there was little effect on decreasing serum TG and TC levels after ginsenoside CK and PPD treatment (Figure 1G–J). In this study, atorvastatin treatment decreased FBG, TC and LDL-C levels in HFD-fed rats, while atorvastatin treatment failed to reduce body weight gain, eWAT/BW, liver/BW, TG and HDL-C levels. To further evaluate the effects of the ginsenoside Rb1, CK, and PPD on alleviating lipid metabolism disorders, HE staining was performed on liver and eWAT in HFD-fed rats. As shown in Supplementary Materials Figure S1D and Figure 1K, ginsenoside Rb1 improved hepatic steatosis and lipid accumulation in eWAT. Similarly, ginsenoside CK and PPD treatment could also slightly reduce hepatic fat droplets and adipocyte size in HFD-fed rats. However, atorvastatin treatment failed to ameliorate adipose deposition and lipid accumulation in liver and eWAT. Furthermore, in the HFD group, serum TNF-α, IL-6, and IL-1β levels of the HFD group were significantly increased compared with the Con group. Ginsenoside Rb1 treatment observably reduced serum TNF-α, IL-6, and IL-1β levels. Ginsenoside CK and PPD treatment decreased serum TNF-α and IL-6 secretion, respectively. In addition, the serum TNF-α level was lower, whereas the serum IL-6 level was higher in the Ato group compared with the HFD group (Figure 1L). These results indicate that ginsenoside Rb1 was more effective than ginsenoside CK and PPD in improving obesity, hyperlipidemia and hepatic steatosis in HFD-fed rats.

### 2.2. Effects of Ginsenoside Rb1, CK, and PPD on the Composition of Gut Microbiota in HFD-Fed Rats

**Figure 2:**
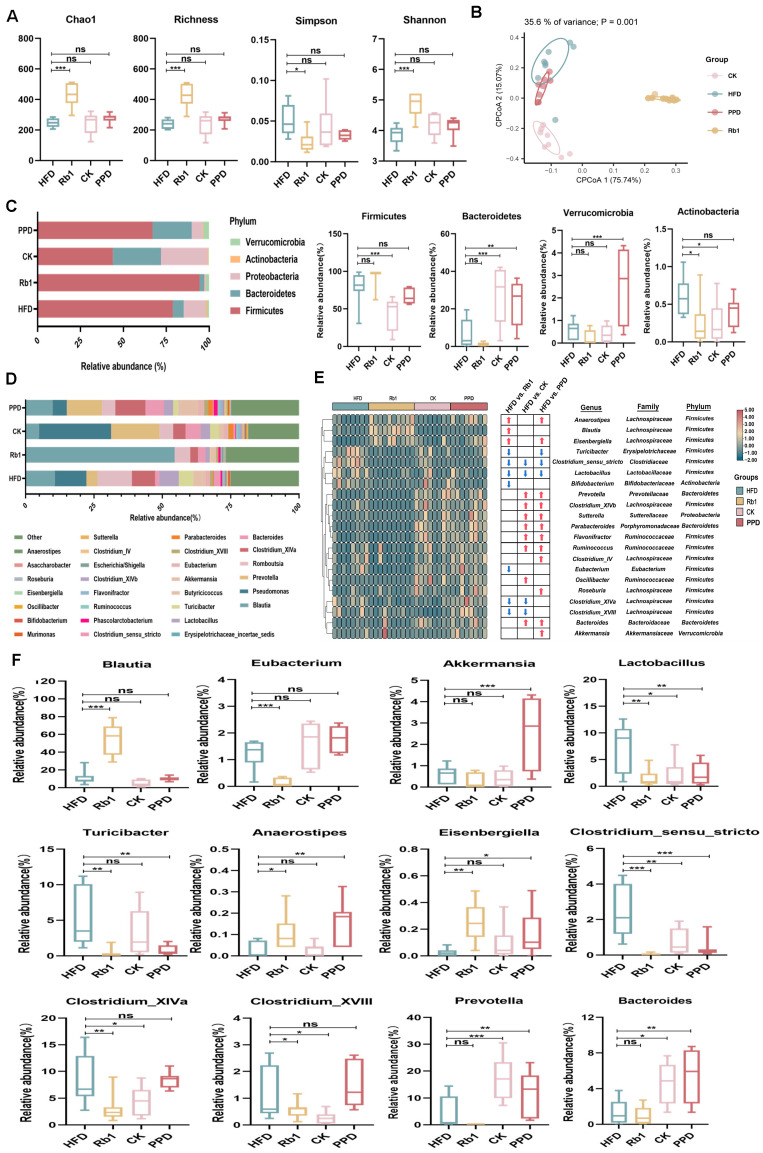
Effects of ginsenoside Rb1, CK and PPD on gut microbiota structure in HFD-fed rats. (**A**) α diversity analysis, (**B**) PCoA analysis, (**C**) phylum-level analysis, (**D**) top 30 genus-level analysis, (**E**) heatmap showing the relative abundance of 21 genera significantly altered after ginsenoside Rb1, CK and PPD treatment, 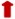 representing more abundance in the Rb1, CK and PPD groups compared with the HFD group, 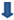 representing less abundance in the Rb1, CK and PPD groups compared with the HFD group, (**F**) change in the relative abundance of representative genera. Values are the means ± SEMs. * *p* < 0.05, ** *p* < 0.01 and *** *p* < 0.001 versus the HFD group, ns means no significantly difference.

As shown in Figure 2A, the Chao1 index, the richness index, Shannon’s index and Simpson’s index estimate species richness and species evenness, which reflect the diversity of microbial communities. After ginsenoside Rb1 treatment, the Chao1 index, the richness index and Shannon’s index were significantly enhanced and Simpson’s index was reduced, while there was no significant difference in the Chao1 index, the richness index, Simpson’s index and Shannon’s index between the CK group, the PPD group and the HFD group. In addition, we observed a distinct clustering of microbiota composition for the HFD group, the Rb1 group, the CK group and the PPD group by UniFrac-based principal coordinates analysis (PCoA) (Figure 2B), indicating that there were significant differences in gut microbiota structure after ginsenoside Rb1, CK, and PPD treatment. Next, we analyzed the relative abundance of gut microbiota at the phylum and genus levels in HFD-fed rats. At the phylum level, the relative abundance of *Actinobacteria* was lower in the Rb1 group. Moreover, the relative abundance of *Firmicutes* and *Actinobacteria* was lower, while the relative abundance of *Bacteroidetes* was higher in the CK group compared with the HFD group. In the PPD group, the relative abundance of *Verrucomicrobia* and *Bacteroidetes* was higher compared with the HFD group (Figure 2C). At the genus level, the relative abundance of the top 30 genera was shown in Figure 2D. Among them, the relative abundance of *Blautia*, *Eisenbergiella*, and *Anaerostipes* was higher, whereas the relative abundance of *Clostridium subcluster XlVa*, *Clostridium cluster XVIII*, *Eubacterium*, *Lactobacillus*, *Turicibacter*, *Bifidobacterium* and *Clostridium-sensu-stricto* was lower in the Rb1 group. The relative abundance of *Clostridium-sensu-stricto*, *Lactobacillus* was observably reduced, while the relative abundance of *Prevotella*, *Clostridium subcluster XlVa*, *Sutterella*, *Parabacteroides*, *Flavonifractor*, *Ruminococcus* and *Bacteroides* was enhanced after ginsenoside CK and PPD treatment (Figure 2E). Particularly, further analysis at the genus level showed that the relative abundance of *Blautia* and *Eubacterium* was changed only after ginsenoside Rb1 treatment. Ginsenoside PPD treatment markedly increased the relative abundance of *Akkermansia* in HFD-fed rats. Ginsenoside Rb1, CK and PPD treatment simultaneously decreased the relative abundance of *Clostridium-sensu-stricto* and *Lactobacillus* in the HFD-fed rats (Figure 2F).

**Figure 3:**
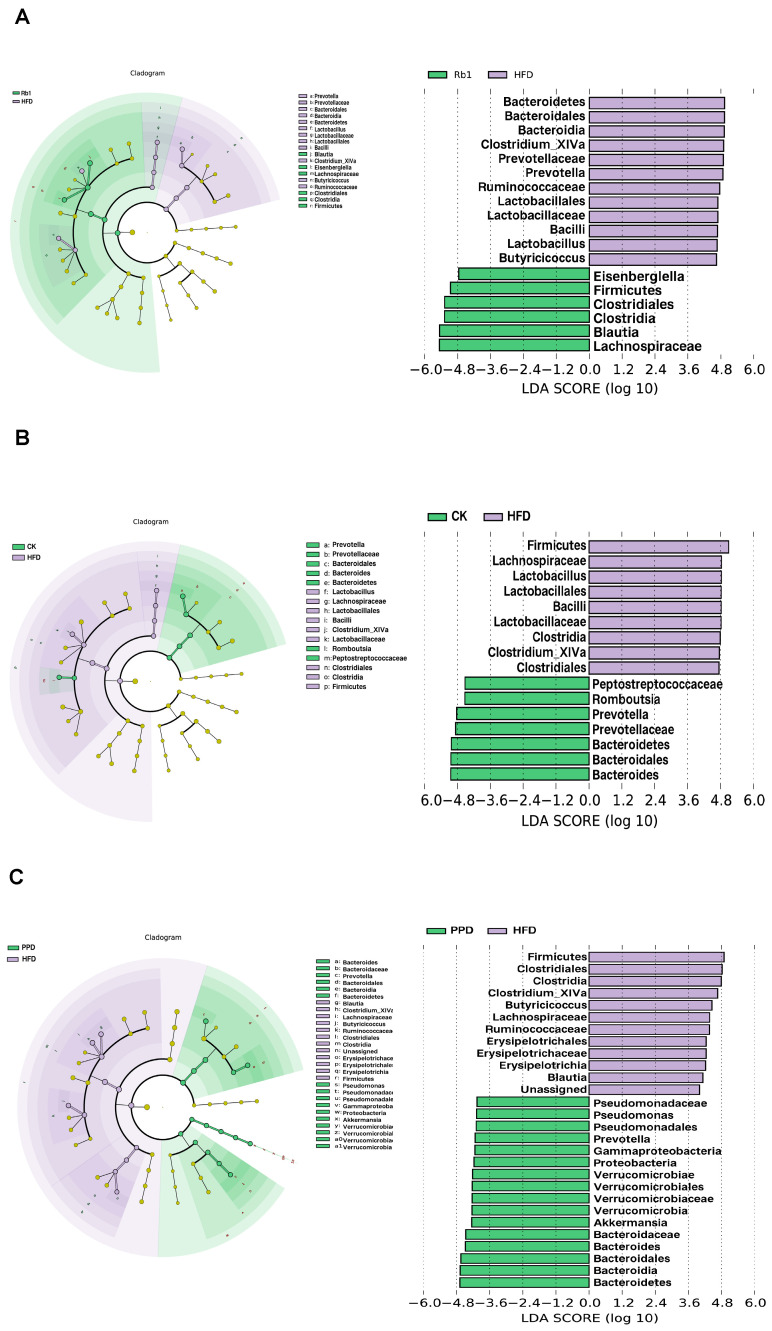
Effects of ginsenoside Rb1, CK and PPD on the gut microbial community in HFD-fed rats. The cladogram of the linear discriminant analysis effect size (LEfSe) analysis for (**A**) Rb1 vs. HFD, (**B**) CK vs. HFD, and (**C**) PPD vs. HFD.

LEfSe analysis also demonstrated significant taxonomic differences at the bacterial genus level among the Rb1 group, the PPD group, the CK group and the HFD group (Figure 3A–C). The relative abundance of *Firmicutes* and *Blautia* was higher, and the relative abundance of *Clostridium subcluster XlVa* and *Lactobacillus* was lower in the Rb1 group compared to the HFD group. In addition, the relative abundance of *Prevotella* and *Bacteroides* was increased and the relative abundance of *Clostridium subcluster XlVa* and *Lactobacillus* was reduced after ginsenoside CK treatment. Similar to the ginsenoside CK treatment, the relative abundance of *Prevotella*, *Bacteroides* and *Akkermansia* was higher and the relative abundance of *Clostridium subcluster XlVa* was lower after ginsenoside PPD treatment. These results were in accordance with the genus-level analysis.

### 2.3. Effects of Ginsenoside Rb1, CK, and PPD on the Function of Gut Microbiota in HFD-Fed Rats

PICRUSt analysis was applied to predict the function of microbial genes involved in the metabolism pathway in the HFD group, the Rb1 group, the CK group, and the PPD group. Compared with the HFD group, multiple pathways related to lipid metabolism were significantly improved after ginsenoside Rb1 treatment, including the superpathway of phospholipid biosynthesis I, fatty acid salvage, fatty acid β-oxidation, CDP-diacylglycerol biosynthesis I, and CDP-diacylglycerol biosynthesis II. In addition, metabolism pathways involved in glycometabolism, including the glycolysis II (from fructose 6-phosphate), D-fructuronate degradation, D-glucuronide and D-glucuronate degradation pathways, were altered in the Rb1 group compared with the HFD group (Figure S2A). Moreover, the fatty acid elongation-saturated, superpathway of fatty acid biosynthesis, GDP-mannose biosynthesis, sucrose degradation III, peptidoglycan maturation and sucrose degradation IV were changed after ginsenoside CK treatment (Figure S2B). Further, lipid IVA biosynthesis was markedly altered after ginsenoside PPD treatment (Figure S2C). Overall, these studies suggested that ginsenoside Rb1, CK, and PPD could regulate the composition and function of gut microbiota in HFD-fed rats in different ways.

### 2.4. Effects of Ginsenoside Rb1, CK, and PPD on FA and Cholesterol Metabolism in HFD-Fed Rats

**Figure 4:**
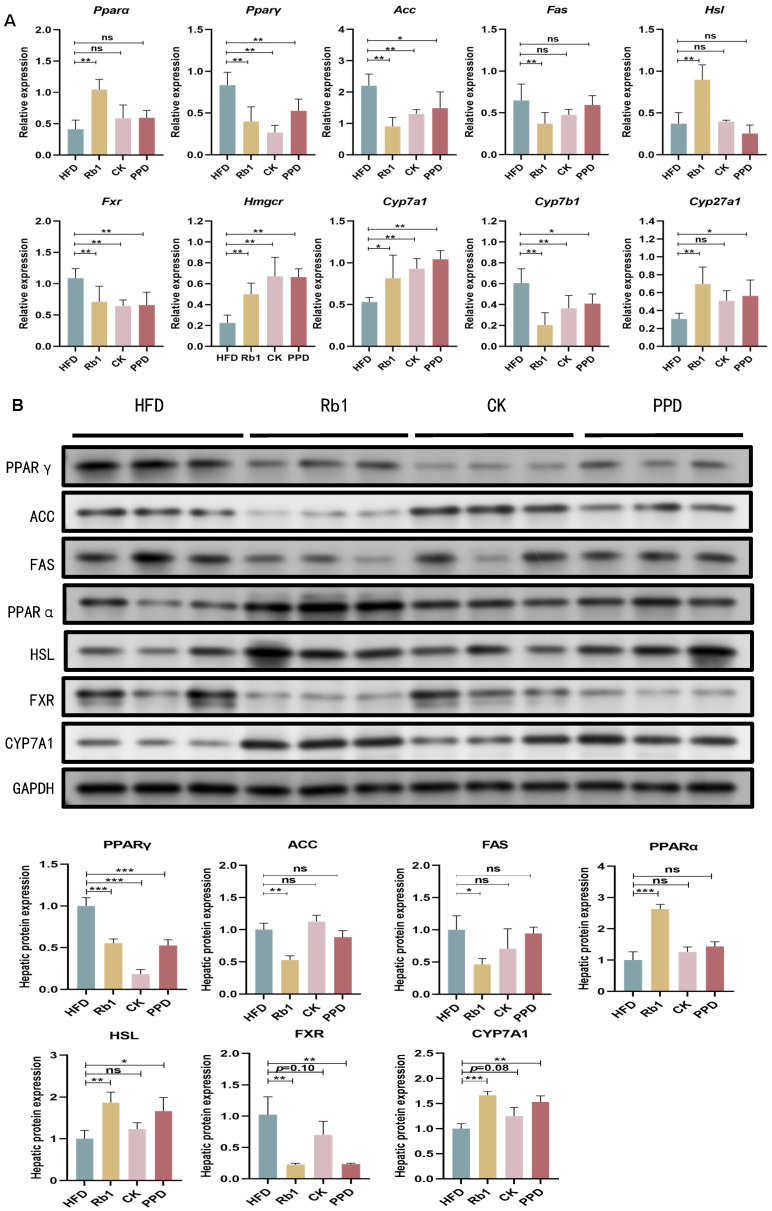
Effects of ginsenoside Rb1, CK and PPD on the relative expression of genes and proteins related to cholesterol metabolism and fatty acid metabolism in HFD-fed rats. (**A**) The relative expression of genes related to cholesterol metabolism and fatty acid metabolism. (**B**) The protein expression of PPARγ, ACC, FAS, PPARα, HSL, FXR and CYP7A1 in liver. Values are the means ± SEMs. * *p* < 0.05, ** *p* < 0.01 and *** *p* < 0.001 versus the HFD group, ns means no significantly difference.

To examine the molecular mechanism of ginsenoside Rb1, CK, and PPD in lipid metabolism, we measured the relative expression of genes related to FA and cholesterol metabolism in the liver, including farnesoid X receptor (*Fxr*), 3-hydroxy-3-methylglutaryl-coenzyme A (CoA) reductase (*Hmgcr*), cytochrome P450 (CYP) isoform 7A1 (*Cyp7a1*), *Cyp7b1*, *Cyp27a1*, perixisome proliferation-activated receptor alpha (*Pparα*), *Pparγ*, acetyl-CoA carboxylase (*Acc*), fatty acid synthase (*Fas*) and hormone-sensitive triglyceride lipase (*Hsl*). As shown in Figure 4A, genes involved in cholesterol synthesis (*Hmgcr*, *Cyp7a1*, *Cyp27a1*) were down-regulated and genes involved in cholesterol catabolism (*Fxr*, *Cyp7b1*) were up-regulated in the HFD group, whereas ginsenoside Rb1, CK, and PPD treatment markedly up-regulated the mRNA relative expression of *Hmgcr*, *Cyp7a1*, and *Cyp27a1* and down-regulated the mRNA relative expression of *Fxr* and *Cyp7b1*. In addition, only ginsenoside Rb1 supplementation significantly down-regulated the relative expression of genes related to FA synthesis *Pparγ*, *Acc* and *Fas* and up-regulated the relative expression of genes related to FA β-oxidation *Pparα* and *Hsl* in HFD-fed rats. Ginsenoside CK and PPD treatment failed to change the relative expression of genes related to FA metabolism. Further, hepatic protein expression related to FA metabolism (ACC, HSL, FAS, PPARα and PPARγ) and cholesterol metabolism (FXR, CYP7A1) was measured among the HFD group, the Rb1 group, the CK group and the PPD group. As shown in Figure 4B, ginsenoside Rb1 supplementation down-regulated the relative expression of PPARγ, ACC and FAS and up-regulated the relative expression of PPARα and HSL in HFD-fed rats. Compared to the HFD group, the relative expression of PPARγ and FXR was lower, whereas the relative expression of CYP7A1 was higher in the CK group. Likewise, the relative expression of PPARγ, FXR was down-regulated, whereas the relative expression of HSL and CYP7A1 was up-regulated in the PPD group. These studies suggested that ginsenoside Rb1, CK, and PPD observably improved cholesterol catabolism (BA metabolism) in HFD-fed rats. Meanwhile, only ginsenoside Rb1 supplementation could alleviate FA metabolism disorder through regulating FA synthesis and β-oxidation.

### 2.5. Effects of Ginsenoside Rb1, CK, and PPD on BA Metabolism in HFD-Fed Rats

**Figure 5:**
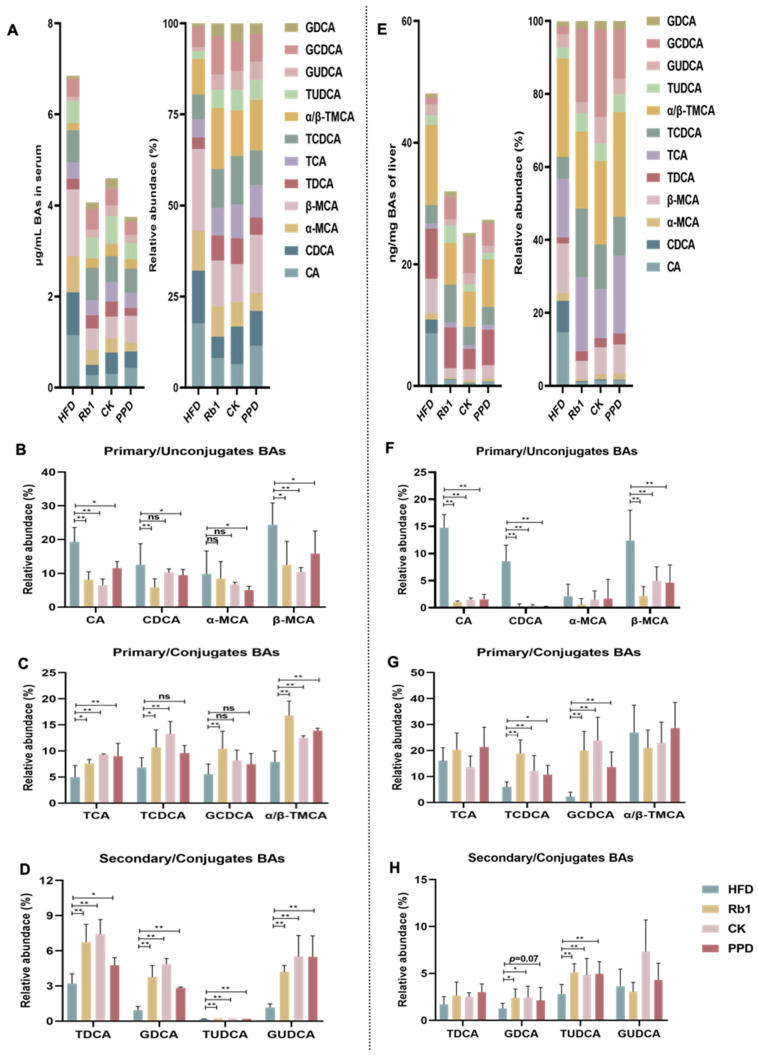
Effects of ginsenoside Rb1, CK and PPD on BA metabolism in HFD-fed rats. (**A**) The total BA level in serum; (**B**) primary/unconjugate BA level in serum; (**C**) primary/conjugate BA in serum; (**D**) secondary/conjugate BA in serum; (**E**) the total BA level in liver; (**F**) primary/unconjugate BA level in liver; (**G**) primary/conjugate BA in liver; (**H**) secondary/conjugate BA in liver. Values are the means ± SEMs. * *p* < 0.05, ** *p* < 0.01 versus the HFD group, ns means no significantly difference.

Furthermore, to further clarify the effect of ginsenoside Rb1, CK, and PPD treatment on BA metabolism, we measured serum and hepatic 12 BA levels by HPLC-MS/MS. As shown in Figure 5A, in serum, the total BA level was significantly decreased in the Rb1, CK, and PPD groups compared with the HFD group. Among them, the relative abundance of primary/unconjugate BA (CA and β-MCA) was lower after ginsenoside Rb1, CK, and PPD treatment. Ginsenoside Rb1 and PPD treatment decreased the relative abundance of CDCA and α-MCA, which was not altered after ginsenoside CK treatment. Further, ginsenoside Rb1 treatment notably enhanced the relative abundance of primary/conjugate BA, including TCA, TCDCA, GCDCA and α/β-TMCA, while ginsenoside PPD treatment did not change the relative abundance of TCDCA and GCDCA. Similarly, ginsenoside CK treatment increased the relative abundance of TCA, TCDCA and α/β-TMCA. Meanwhile, the relative abundance of secondary/conjugate BA, including TDCA, GDCA, TUDCA and GUDCA, was significantly reduced in the Rb1, CK and PPD groups compared with the HFD group. In liver, the BA profile was similar to that in serum. The total BA level was significantly decreased after ginsenoside Rb1, CK and PPD treatment. The relative abundance of primary/unconjugate BA, CA, CDCA and β-MCA was lower in the Rb1 group, the CK group and the PPD group than that in the HFD group. Furthermore, TCDCA, GCDCA, GDCA and TUDCA, belonging to conjugate BA, were observably increased after ginsenoside Rb1, CK and PPD treatment. Collectively, these results indicated that ginsenoside Rb1, CK, and PPD treatment could decrease the accumulation of hepatic and serum total BAs and regulate the profile of the BA pool in HFD-fed rats.

### 2.6. Correlation Analysis of Gut Microbiota and BA Metabolism

**Figure 6:**
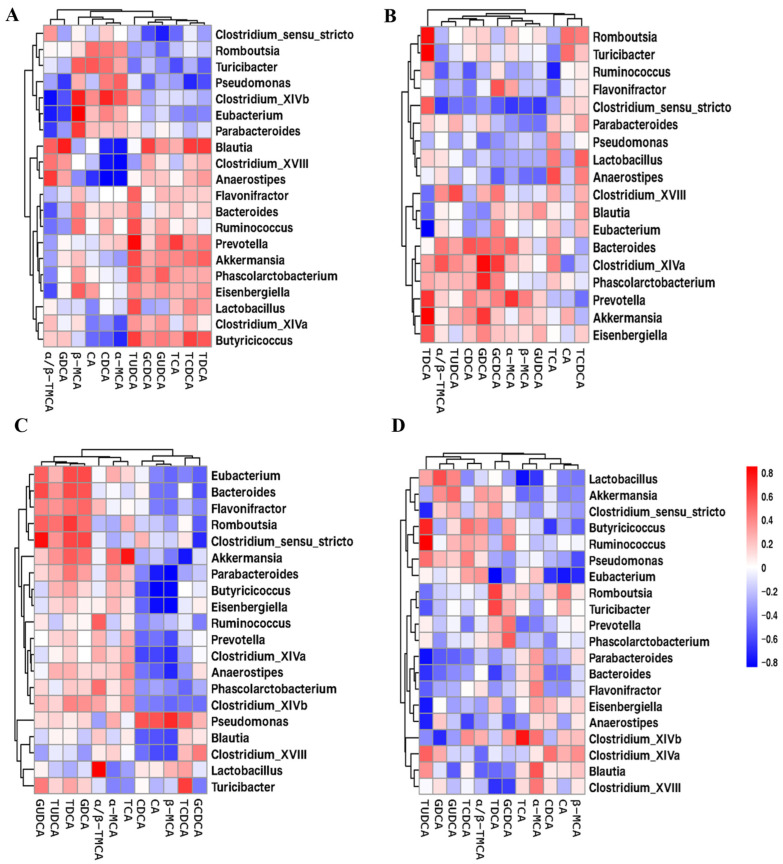
The heatmap showed Pearson’s correlation coefficient with *p* < 0.05 between key gut microbes and BAs in the HFD, Rb1, CK and PPD groups. (**A**) The correlation between the top 20 genus and serum BAs in the HFD group, (**B**) the correlation between the top 18 genus and serum BAs in the Rb1 group, (**C**) the correlation between the top 20 genus and serum BAs in the CK group, and (**D**) the correlation between the top 20 genus and serum BAs in the PPD group. Red cells represent positive correlation and blue cells represent negative correlation.

To clarify the correlation between gut microbiota and the levels of BAs in serum, Spearman correlation analysis was further performed among the four groups. The serum 12 BAs were correlated with the changes in the top 20 genus. As shown in Figure 6A, in the HFD group, *Clostridium_sensu_stricto*, *Romboutsia*, *Turicibacter*, *Pseudomonas*, *Clostridium_XlVb*, and *Eubacterium* were negatively correlated with conjugate BA (TUDCA, GCDCA, GUDCA, TCA, TCDCA and TDCA), positively correlated with the primary/unconjugate BA (CDCA, CA, α-MCA, β-MCA). Conversely, *Blautia*, *Anaerostipes*, *Flavonifractor*, *Prevotella*, *Akkermansia*, *Phascolarctobacterium*, *Eisenbergiella* and *Butyricicoccus* were positively correlated with conjugate BA (TUDCA, GCDCA, GUDCA, TCA, TCDCA and TDCA), and *Blautia*, *Clostridium_XVIII*, *Anaerostipes*, *Clostridium_XlVa* and *Butyricicoccus* were negatively correlated with primary/unconjugate BA, including α-MCA and CDCA. In Rb1 group, most BAs (except CA and TCDCA) were positively correlated with *Bacteroides*, *Clostridium_XlVa*, *Phascolarctobacterium*, *Prevotella*, *Akkermansia* and *Eisenbergiella*, and negatively correlated with *Clostridium_sensu_stricto*, *Pseudomonas*, *Lactobacillus* and *Anaerostipes* (Figure 6B). Moreover, primary BAs (CA, CDCA, β-MCA, TCDCA and GCDCA) were negatively correlated with most genera (except *Pseudomonas* and *Lactobacillus*), whereas GUDCA, TUDCA, TDCA and GDCA were positively correlated with *Eubacterium*, *Bacteroides*, *Flavonifractor*, *Romboutsia*, *Clostridium_sensu_stricto*, *Akkermansia* and *Parabacteroides* in the CK group (Figure 6C). In addition, *Lactobacillus*, *Akkermansia*, *Clostridium_sensu_stricto*, *Butyricicoccus* and *Pseudomonas* were negatively correlated with primary/unconjugate BA (CDCA, CA, α-MCA, β-MCA), and positively correlated with GDCA and GUDCA. On the contrary, *Parabacteroides*, *Bacteroides*, *Flavonifractor*, *Eisenbergiella* and *Clostridium_XlVb* were negatively correlated with TUDCA, GDCA and GUDCA, and positively correlated with α-MCA and TCA (Figure 6D). These results suggested that key-phylotypes in the gut microbiota after ginsenoside Rb1, CK, and PPD treatment were strongly correlated with the serum BAs in HFD-fed rats.

## 3. Discussion

Many studies have reported that the gut microbiota has been shown to be associated with cardiovascular disease (CVD) development such as obesity, type 2 diabetes (T2DM), metabolic syndrome, and hyperlipidemia. Dietary intervention plays critical roles in maintaining a healthy cardiovascular function and contributes to alleviating insulin sensitivity and lipid metabolism dysfunction through regulating gut microbiota composition [19,20]. Ginsenoside has been attracting attention as a dietary phytochemical, which decrease TC and TG levels in HFD-induced mice [21]. However, whether the beneficial effects of ginsenoside Rb1, CK, and PPD treatment has HFD-induced hyperlipidemia could be attributed to the regulation of gut microbiota and BA metabolism has been rarely reported. In this study, we aimed to investigate the effects of ginsenoside Rb1, CK, and PPD treatment on hyperlipidemia in HFD-fed rats and further explore their differences and underlying mechanisms by focusing on the gut microbiota and BA metabolism. Specifically, ginsenoside Rb1 treatment reduced body weight gain and serum TC and TG levels, and alleviated hepatic steatosis and inflammation, whereas ginsenoside CK and PPD treatment had no such effect. Serum FBG and LDL-C levels were significantly reduced and the serum HDL-C level was not altered after ginsenoside Rb1, CK, and PPD treatment. Moreover, ginsenoside Rb1, CK, and PPD treatment changed the structure and function of gut microbiota through different ways, which were closely connected with BA metabolism in HFD-fed rats. 

Previous studies have indicated that ginsenoside Rb1 supplementation improved HFD-induced insulin resistance and obesity in mice [22]. Similarly, ginsenoside CK and PPD was shown to reduce body weight and fat accumulation and improve sensitivity to insulin in mice [23,24]. Our results were consistent with previous studies that ginsenoside Rb1 treatment observably reduced body weight, body weight gain, eWAT/BW and liver/BW in HFD-fed rats (Figure 1B–E). However, ginsenoside CK and PPD failed to affect the increased body weight after HFD treatment in rats. In addition, ginsenoside Rb1 improved hyperlipidemia through decreasing serum TC, TG and LDL-C levels, while ginsenoside CK and PPD treatment partly alleviated hyperlipidemia by reducing the LDL-C level. There was no significant difference in the HDL-C level after ginsenoside Rb1, CK and PPD supplementation (Figure 1G–J). Moreover, ginsenoside Rb1 treatment could ameliorate hepatic steatosis though reducing adipose deposition in liver and eWAT, which was better than ginsenoside CK and PPD treatment (Figure 1K). Statins, such as 3-hydroxy-3-methyl-glutaryl coenzyme A (HMG-CoA) reductase inhibitor, suppressed the cholesterol biosynthetic pathway, resulting in decreased TC and LDL-C levels in serum [25]. In our study, atorvastatin treatment did not reduce body weight or relieve hepatic steatosis, which could improve hyperlipidemia by reducing serum TC and LDL-C levels in HFD-fed rats. 

A growing body of research has underscored the pivotal role of inflammatory cytokines in the initiation and advancement of hyperlipidemia and obesity. Lai et al. have elucidated that various inflammatory processes exhibit a strong correlation with hyperlipidemia and its concomitant cardiovascular diseases (CVDs). Notably, the circulating levels of cytokines associated with inflammation, namely IL-1β, IL-6, and TNF-α, were found to be significantly higher in patients afflicted with CVDs [26]. Furthermore, a link between obesity and elevated serum levels of inflammatory cytokines has been established. The surplus of macronutrients within adipose tissues acts as a catalyst for the secretion of inflammatory mediators, such as TNF-α and IL-6, while concurrently diminishing the production of adiponectin. This imbalance fosters a pro-inflammatory state and induces oxidative stress [27]. The escalation of cytokines in individuals with obesity is implicated in the progression of several disorders, including cardiovascular disease, hypertension, and insulin resistance [28].

Targeting inflammatory pathways offers a promising strategy for managing obesity and mitigating the risk of cardiovascular diseases. Ginsenosides, in particular, have been identified as potent agents in dampening inflammation. Oral administration of ginsenosides has been shown to significantly reduce inflammatory markers. Specifically, ginsenoside Rb1 has emerged as a potential anti-inflammatory agent, demonstrating the ability to inhibit the activation of NF-κB, a pivotal regulator of inflammation and a key factor in controlling TNF-α production in LPS-activated mouse peritoneal macrophages [29]. Furthermore, Compound K (CK) exerts its anti-inflammatory effects primarily by diminishing the levels of inducible nitric oxide synthase (iNOS), cyclooxygenase (COX)-2, and pro-inflammatory cytokines [29,30,31]. CK achieves this by down-regulating the activity of IRAK-1, MAPKs, IKK-α, and NF-κB in the peritoneal macrophages of LPS-treated mice [32]. In our study, we observed a notable reduction in serum TNF-α, IL-6, and IL-1β levels following treatment with ginsenoside Rb1, in comparison to the high-fat diet (HFD) group. Additionally, treatments with ginsenoside CK and PPD individually led to a decrease in serum TNF-α and IL-6 levels, respectively (Figure 1L). Our findings confirm the anti-inflammatory properties of ginsenoside.

The importance of the gut microbiota in human health has been revealed. The imbalance of gut microbiota was related to many metabolic diseases, such as hyperlipidemia, non-alcoholic liver disease and atherosclerosis [33,34]. The effect mechanism of microbiota-related metabolites such as BAs, lipopolysaccharide (LPS), and short-chain fatty acids (SCFAs) in the regulation of hyperlipidemia has been reported [35]. In our study, ginsenoside Rb1 improved the diversity of gut microbiota through increasing the Chao1 index, the richness index and Shannon’s index, whereas ginsenoside CK and PPD failed to change the diversity index in HFD-fed rats (Figure 2A). Furthermore, *Blautia*, as a dominant genus in the gut microbiota, has revealed a negative correlation with obesity-related metabolic disorders [36]. After ginsenoside Rb1 treatment, the relative abundance of *Blautia* was significantly enhanced (58.75%) in HFD-fed rats (Figure 2F). The genus *Akkermansia*, belonging to the *Verrucomicrobia* phylum, is considered to be a promising candidate as a probiotic, which regulates metabolic functions and protects mice from high-fat diets [37,38]. The relative abundance of *Akkermansia* was increased after ginsenoside PPD treatment. In addition, some studies indicated that *Lactobacillus* have shown inhibitory effects on HFD-induced obesity, whereas Aline et al.’s study indicated that the dysbiosis of gut microbiota seems be related to increases in *Lactobacillus* [19,39]. *Clostridium_sensu_stricto* is regarded as the true *Clostridium* genus, which is associated with lipid metabolism disorders and insulin resistance [40]. Our results indicated that the relative abundance of *Lactobacillus* and *Clostridium_sensu_stricto* was decreased after ginsenoside Rb1, CK, and PPD treatment. Meanwhile, previous studies had shown that the relative abundance of *Clostridium_XIVa* was enhanced in feces of 34 obese subjects with metabolic syndrome than without metabolic syndrome [41] and *Clostridium_XVIII* was positively related to obese phenotypes in mice [42]. Ginsenoside Rb1 and CK treatment reduced the relative abundance of *Clostridium_XIVa* and *Clostridium_XVIII* in HFD-fed rats. In addition, *Prevotella* and *Bacteroides* were the dominant genera after ginsenoside CK and PPD treatment. Previous studies have shown that ginsenoside Rb1 has only three more molecules of glucose (Glc) than ginsenoside CK, which is transformed into ginsenoside CK and PPD under the action of *Prevotella* and *Bacteroides* by removing Glc at C-3 [17,43]. In our study, the relative abundance of *Prevotella* and *Bacteroides* was significantly increased after ginsenoside CK and PPD treatment, while ginsenoside Rb1 treatment had no similar effect. These results suggested that ginsenoside Rb1, CK, and PPD selectively increased and reduced intestinal bacterial genera, which contributed to the anti-hyperlipemia effects in HFD-fed rats.

It is well known that *lactobacilli* are common probiotics that play a major role in ameliorating several metabolic disorders. However, our study found a reduction in *Lactobacillus* abundance following treatment with all three ginsenosides. This finding is indeed intriguing, considering the well-documented beneficial effects of various *Lactobacillus* strains on hyperlipidemia [44,45,46]. While many studies highlight the positive effects of *Lactobacillus* on hyperlipidemia, the interaction between ginsenosides and *Lactobacillus* is less well understood. Some research suggests that ginsenosides can have prebiotic-like effects, promoting the growth of beneficial gut bacteria [47]. However, these effects may not be uniform across all bacterial strains, and our findings underscore the need for a nuanced understanding of how specific ginsenosides interact with specific strains of *Lactobacillus*. In addition, it is also possible that ginsenosides may exert lipid-lowering effects through mechanisms independent of *Lactobacillus* modulation. 

Concomitant with gut microbial composition alteration, we observed a regulation in bacterial gene functions after ginsenoside Rb1, CK, and PPD supplementation. Through PICRUSt KEGG function analysis, we found that the gene abundance involved in lipid metabolism pathways (the superpathway of phospholipid biosynthesis I, fatty acid salvage, fatty acid β-oxidation, CDP-diacylglycerol biosynthesis I, and CDP-diacylglycerol biosynthesis II) and glycometabolism (glycolysis II (from fructose 6-phosphate), D-fructuronate degradation, D-glucuronide and D-glucuronate degradation pathway) changed after ginsenoside Rb1 supplementation. Further, ginsenoside CK intervention altered glycometabolism pathways (sucrose degradation III, peptidoglycan maturation and sucrose degradation IV) to a greater extent compared to lipid metabolism pathways (fatty acid elongation-saturated and superpathway of fatty acid biosynthesis). Only the genes involved in the lipid IVA biosynthesis pathway were enriched in the PPD group compared with the HFD group (Figure S2A–C). 

Next, we measured gene expression involved in lipid metabolism pathways in the HFD group, the Rb1 group, the CK group and the PPD group. Peroxisome proliferator-activated receptors (PPARs) have been considered as integrators of inflammatory and metabolic signaling networks [48]. PPARα controls lipid oxidation by regulating the expression of genes related to fatty acid transport and β-oxidation, thus reducing plasma levels of TG [49]. In contrast, PPARγ accelerates fatty acid uptake, TG formation and storage in lipid droplets, resulting in increased insulin sensitivity and glucose metabolism. The activation of PPARγ continues to regulate the downstream genes ACC1 and FAS, thus accelerating fatty acid synthesis [50]. In adipose tissues, stored TGs were hydrolyzed to free fatty acids by HSL [51]. In our study, the relative expression of *Pparα* and *Hsl* was up-regulated and the relative expression of *Pparγ*, *Acc* and *Fas* was down-regulated after ginsenoside Rb1 treatment. Likewise, hepatic protein expression of PPARα and HSL was higher and that of PPARγ, ACC and FAS was lower in the Rb1 group compared with the HFD group. These results indicated that ginsenoside Rb1 could improve lipid metabolism through regulating the PPARγ/ACC/FAS signaling pathway and stimulating FA β-oxidation in HFD-fed rats, consistent with the function analysis of gut microbiota. However, ginsenoside CK and PPD treatment down-regulated the relative expression of PPARγ, which failed to alter the relative expression of PPARα, HSL ACC and FAS. This result was in agreement with our studies that ginsenoside Rb1, but not ginsenoside CK and PPD, could reduce serum TC and TG levels in HFD-fed rats. Furthermore, FXR, as a BA synthesis and enterohepatic circulation mediator, plays a key role in BAs and cholesterol homeostasis, which regulated the rate-limiting enzyme (CYP7A1) for BA synthesis [52]. HMGCR, as the rate-limiting enzyme of cholesterol biosynthesis, contributes to cholesterol biosynthesis and uptake [53]. However, the relative expression of *Hmgcr* was enhanced in the Rb1, CK and PPD groups compared with the HFD group, which still needs further research. Notably, whether ginsenoside Rb1 or CK and PPD treatment, gene and protein relative expression of FXR and CYP7A1 were significantly changed in HFD-fed rats. Therefore, on the one hand, we assumed that ginsenoside Rb1 treatment inhibited lipogenesis via the PPARγ/ACC1/FAS pathway and promoted FA β-oxidation by down-regulating the mRNA expression of PPARα and HSL. On the other hand, ginsenoside Rb1, CK, and PPD could act as potent FXR agonists that ameliorate the TC level through regulating BA metabolism. 

Further, to clarify the regulatory effect of ginsenoside Rb1, CK, and PPD treatment on BA metabolism, we measured hepatic and serous 12 BA levels in HFD-fed rats. The hepatic and serous total BA levels were markedly decreased after ginsenoside Rb1, CK, and PPD treatment in HFD-fed rats. Previous studies have indicated serum total BAs can be used to evaluate hepatic dysfunction and diagnose the various hepatobiliary diseases [54]. Increased serum BA levels induced metabolic dysfunctions and positively correlated obesity and type 2 diabetes [55,56]. In our study, compared with the HFD group, the serum primary/unconjugate BA (CA and β-MCA) level was lower in the Rb1 group, the CK group and the PPD group. Further, only ginsenoside PPD treatment could reduce the serum α-MCA levels and there was no significant difference in hepatic α-MCA levels among these groups. The ginsenoside Rb1 and PPD treatment decreased the serum CDCA level, whereas ginsenoside CK had no such effect. In liver, the primary/unconjugate BA (CA, CDCA and β-MCA) were markedly decreased after ginsenoside Rb1, CK, and PPD treatment, which was consistent with serum primary/unconjugate BA profile. FXR regulates hepatic BA synthesis via a negative feedback mechanism. Activated FXR suppresses the expression of CYP7A1 and thus inhibits hepatic BA synthesis. CDCA and CA were shown to be the agonists of FXR function, inhibiting BA synthesis [57]. In the HFD group, increased CA and CDCA levels could activate FXR and inhibit hepatic BA synthesis, leading to more cholesterol accumulation in the liver. After ginsenoside Rb1, CK, and PPD treatment, the hepatic and serous primary/unconjugate BA (CA, CDCA and β-MCA) were decreased in the HFD-fed rats, indicating that ginsenoside Rb1, CK, and PPD treatment promoted hepatic BA synthesis and reduced hepatic cholesterol levels. Furthermore, serum conjugate BA, including TCA, α/β-TMCA, TDCA, GDCA, TUDCA and GUDCA, were enhanced in the Rb1, CK, and PPD groups. Likewise, the hepatic conjugate BA (TCDCA, GUDCA, TDCA, GDCA, TUDCA and GUDCA) increased after ginsenoside Rb1, CK, and PPD treatment. Jiang et al. research suggested that alteration of the gut microbiota changed BA composition, notably alteration of conjugated BAs that regulate the intestinal FXR [58]. In liver, ginsenoside Rb1, CK, and PPD contributed to the conjugation of primary BAs with taurine (T) and glycine (G), thereby increasing BA hydrophilicity, reducing the hepatotoxicity of BAs and inhibiting cholestatic liver injury [59]. Our results suggested that ginsenoside Rb1, CK, and PPD regulated BA enterohepatic circulation through enhancing hepatic FXR-CYP7A1 signaling, resulting in decreased total BAs and BA profile alteration in the liver and serum. 

Notably, the gut microbiota has been demonstrated to be a central regulator in BA metabolism. BAs are metabolized by enzymes derived from gut microbiota and play a critically important role in balanced lipid metabolism, insulin sensitivity and innate immunity [57]. In our study, the correlation between serum BAs and gut microbiota was analyzed after ginsenoside Rb1, CK, and PPD treatment. We found that ginsenoside Rb1, CK, and PPD treatment significantly reduced the abundance of BSH-producing *Clostridium* spp., which resulted in increased levels of conjugated BAs [60]. Furthermore, GUDCA, TUDCA, TDCA and GDCA were positively correlated with *Bacteroides* and *Akkermansia* in the Rb1 and CK groups (Figure 6B,C). In addition, *Parabacteroides*, *Bacteroides*, *Flavonifractor*, *Eisenbergiella* and *Clostridium_XlVb* were negatively correlated with TUDCA, GDCA and GUDCA, and positively correlated with α-MCA and TCA (Figure 6D). This notion was supported by previous research [61].

## 4. Materials and Methods

### 4.1. Animal Study Design

Four-week-old male SD rats were purchased from Beijing Vital River Laboratory Animal Technology Co., Ltd. (Beijing, China). They were raised under specific pathogen-free (SPF) conditions in an animal facility with a temperature of 20–25 °C, a relative humidity of (55 ± 15)%, and an air exchange rate of 10–20 times/h. Artificial lighting was controlled to provide a 12 h day/night cycle. Four rats were placed in each cage and had free access to food and water. After 7 days of acclimation, the rats were randomly divided into two groups (*n* = 10 and *n* = 50) and a fed standard diet (1010086) and a high-fat diet (XT19004), respectively. The standard diet and high-fat diet were purchased from Jiangsu Synergetic Biotechnology Co., Ltd. (Nanjing, China). Ginsenoside CK (CAS No. 39262-14-1) was purchased from BBI Life Sciences (Shanghai, China). Ginsenoside Rb1 (CAS No. 41753-43-9) was purchased from Chengdu Pusen Biotechnology Co., Ltd. (Chengdu, China). Ginsenoside PPD (CAS No. 30636-90-9) was provided by Shanghai GsynBioT Bio-Technology Co., Ltd. (Shanghai, China). All three ginsenosides were dissolved in 0.5% CMC-Na and were orally administered by gavage daily.

After 15 days of standard diet and high-fat diet treatment, rats were divided into six groups: (1) the Con group (n = 10), fed with a standard diet and administered with 2 mL/kg∙BW of saline via gavage; (2) the HFD group (n = 10), fed with a high-fat diet and administered with 2 mL/kg∙BW of saline via gavage; (3) the Ato group (n = 10), fed with a high-fat diet and administered with atorvastatin at a dose of 10 mg/kg∙BW via gavage; (4) the Rb1 group (n = 10), fed with a high-fat diet and administered with ginsenoside Rb1 at a dose of 60 mg/kg∙BW via gavage; (5) the CK group (n = 10), fed with a high-fat diet and administered with ginsenoside CK at a dose of 60 mg/kg∙BW via gavage; (6) the PPD group (n = 10), fed with a high-fat diet and administered with ginsenoside PPD at a dose of 60 mg/kg·BW via gavage. All of these treatments were orally administered and lasted for 30 days. The body weight (BW) of rats was measured every 2 days. After 30 days, animals were euthanized by CO_2_ asphyxiation. Dissected tissues, feces and serum were immediately frozen in liquid nitrogen and stored at −80 °C for further analysis.

### 4.2. Biochemical Analysis

Serum total cholesterol (TC), triglyceride (TG), high-density lipoprotein (HDL-C) and low-density lipoprotein (LDL-C) were measured using commercial assay kits according to the manufacturer’s instructions (Nanjing Jiancheng Bioengineering Institute (Nanjing, China)). Serum tumor necrosis factor-α (TNF-α), interleukin-6 (IL-6) and interleukin-1β (IL-1β) were measured using commercial enzyme-linked immunosorbent assay kits (Beijing Baoruyi Biotechnology Co., LTD Co. Ltd., Beijing, China).

### 4.3. Histological Analysis

Liver and epididymal white adipose tissue (eWAT) were fixed in 10% formalin and stained with hematoxylin and eosin. The slices were examined under a light microscope (Olympus, BH-2, Tokyo, Japan) for tissue pathological analysis.

### 4.4. Real-Time Quantitative PCR (RT-qPCR) Analysis

Total RNA was extracted from liver tissue using a RNAprep Pure Tissue Kit (TianGen, Beijing, China) and the RNA samples concentration was measured by Nanodrop one (Thermo Scientific, Waltham, MA, USA). The RNA was then converted to cDNA using a Fastking RT Kit (TianGen, Beijing, China). Reverse transcription and quantitative PCR were performed using SuperReal PreMix Plus Kit (TianGen, Beijing, China) and LightCycler^®^96 System (Roche, Basel, Switzerland). The target genes’ expression was analyzed using the ΔΔCt method with GAPDH as the internal reference gene. The primers used are listed in Supplementary Materials Table S1.

### 4.5. Western Blot Analysis

Liver tissues were homogenized in RIPA buffer (P0013B, Beyotime, Shanghai, China) with protease and phosphatase inhibitors. Proteins were separated on SDS-PAGE and then transferred to PVDF membranes. TBS-Tween containing 5% skimmed milk powder was used to immerse the PVDF membranes at room temperature on a shaker for 2 h. Primary antibodies were incubated overnight at 4 °C. After blotting, the membranes were washed and incubated with secondary antibody for 1 h at room temperature. Target proteins were normalized against β-actin. The protein bands were examined by using the ChemiDoc MP Imaging System (Bio-Rad, Hercules, CA, USA) and the intensities of these proteins were analyzed by the Image J 1.5.4 software (Madison, WI, USA).

### 4.6. Extraction and Sequencing of Fecal DNA Using 16S rRNA Gene

According to the manufacturer’s protocol, the Tiangen Magnetic Stool DNA Kit was used to extract total genomic DNA from fecal samples. The V3–V4 hypervariable regions of 16S rRNA genes were amplified with specific primers. The PCR products were separated on a 2% agarose gel and visualized under UV light. Samples with bright main bands between 400 and 450 bp were selected for further experiments. The PCR products were purified using the Qiagen Gel Extraction Kit (Qiagen, Hong Kong) and then subjected to library preparation using the TruSeq^®^ DNA PCR-Free Sample Preparation Kit (San Diego, CA, USA) according to the manufacturer’s recommendations, including the addition of index codes. The quality and quantity of the libraries were assessed using the Qubit@ 2.0 Fluorometer (Thermo Scientific) and the Agilent (Santa Clara, CA, USA) Bioanalyzer 2100 system. Finally, the libraries were sequenced on the Illumina NovaSeq6000 platform, generating paired-end reads of 250 bp. 16S rRNA gene sequencing analyses were performed using Usearch (v11.0.1). Alpha diversity (number of observed OTUs, the Chao1 index, the richness index, Shannon’s index, Simpson’s index) was visualized and principal coordinates analysis (PCoA, Tucson, AZ, USA) was performed to measure beta diversity. Linear discriminant analysis Effect Size (LEfSe) was used to analyze the biomarkers within different groups. PICRUSt2 was performed to predict the gut microbiota function. 

### 4.7. UHPLC-MS/MS Analysis

The BA concentrations in the serum were quantified using the ACQUITY Ultra performance liquid chromatography (UPLC) quaternary system coupled with a 6500 QTRAP mass spectrometer (AB SCIEX, Toronto, ON, Canada). A Waters UPLC BEH C18 (2.5 μm × 100 mm, 2.1 mm) was used for the separation. The mobile phase consisted of solvent A (water:formic acid (100:0.1, *v*/*v*)) and solvent B (methanol: formic acid (100:0.1, *v*/*v*)). The gradient was as follows: 60–65% B from 0 to 13 min, 65–80% B from 13 to 28 min, 80–90% B from 28 to 28.5 min, 90–100% B from 28.5 to 30 min, and 100–60% B from 30 to 40 min. The column temperature was 50 °C and the sample injection volume was 2 μL. Optimization of multiple reaction monitoring (MRM) parameters was performed on a ESI-QqQ-MS operated in the negative ion mode for BA analysis. The optimal MS conditions were as follows: ion spray voltage: 4500 V; temperature: 550 °C; curtain gas (CUR):40; Ion Source Gas1 (Gas1):55; Ion Source Gas2 (Gas2):55. Acquired data were processed using Analyst Software (version 1.6.1, AB Sciex).

### 4.8. Statistical Analysis

Graphpad Prism 9.2.0 software (GraphPad Software Inc., San Diego, CA, USA) was used to perform the statistical analyses and visualize the data. All data were presented as the mean ± SD. All *p* values were corrected for Dunnett’s multiple comparisons test and subsequent significant differences established were recorded as follows: * *p* < 0.05, ** *p* < 0.01 and *** *p* < 0.01. Correlations were assessed with the Spearman nonparametric rank test. 

## 5. Conclusions

In conclusion, these findings revealed that ginsenoside Rb1 significantly attenuated HFD-induced body weight gain and improved hepatic steatosis and hyperlipidemia, whereas ginsenoside CK and PPD did not show a similar effect. Meanwhile, gut microbiota sequencing studies indicated that ginsenoside Rb1, CK and PPD treatment could reshape the structure and function of gut microbiota through regulating the relative abundance of different genera in HFD-fed rats, including *Blautia*, *Akkermansia*, *Clostridium_XIVa* and *Clostridium_XVIII*, *Lactobacillus* and *Clostridium_sensu_stricto*. Furthermore, ginsenoside Rb1 supplement improved lipid metabolism through regulating the PPARγ/ACC/FAS signaling pathway and stimulating FA β-oxidation in HFD-fed rats, which was consistent with the function analysis of gut microbiota. In addition, ginsenoside Rb1, CK and PPD treatment all regulated BA metabolism through the FXR/CYP7A1 signaling pathway, decreasing total BA levels and altering BA enterohepatic circulation in HFD-fed rats. Our findings provide evidence supporting the beneficial effects of ginsenoside Rb1, CK and PPD intake in ameliorating hyperlipidemia in HFD-fed rats.

## Data Availability

The data presented in this study are available in article and Supplementary Materials.

## Supplementary Materials

The following supporting information can be downloaded at: https://www.mdpi.com/article/10.3390/molecules29051108/s1.
