# Supplementary material for: Ginsenoside Rb1, Compound K and 20(S)-Protopanaxadiol Attenuate High-Fat Diet-Induced Hyperlipidemia in Rats via Modulation of Gut Microbiota and Bile Acid Metabolism
Source: Molecules. 2024 Mar 1;29(5):1108. doi: 10.3390/molecules29051108 (PMC10934602; doi:10.3390/molecules29051108)
Supplement: Supplementary file 1 [file molecules-29-01108-s001.zip › molecules-2881478-supplementary.pdf]

Supplementary Table S1. Primers for RT-qPCR.

Genes	Forward primer	Reverse primer
PPARα	TGAAAGATTCGGAAACTGC	TTCCTGCGAGTATGACCC
PPARγ	TGGAGCCTAAGTTTGAGTT	CAATCTGCCTGAGGTCTG
ACC	ACGCTATCATCAGTGCATA	TGCGATAGTAGTCACGTAT
FAS	AAACCGACAACAACTGCT	TCTAAACCATGCCCTTCA
HSL	CGCCTTACGGAGTCTATGC	GCTGTCTGATGGCTCTGAGTT
FXR	TCATCCTCTCTCCAGACAGACA	AAATGCTGAGGGTTCTCGGG
HMGCR	CACAGAATGTGGGGAGTT	GCTGAGGTAGAAGGTTGG
CYP7A1	GCTGGCTGAGGGATTGAA	AAAGGTGGAGAGCGTGTC
CYP7B1	CATCATCTTGGCTTGCTC	ATTCCAGGTCCTTTCTTT
CYP27A1	TTTCAAAGAACCCAGAGA	CGTAGTGGCATAACACAA

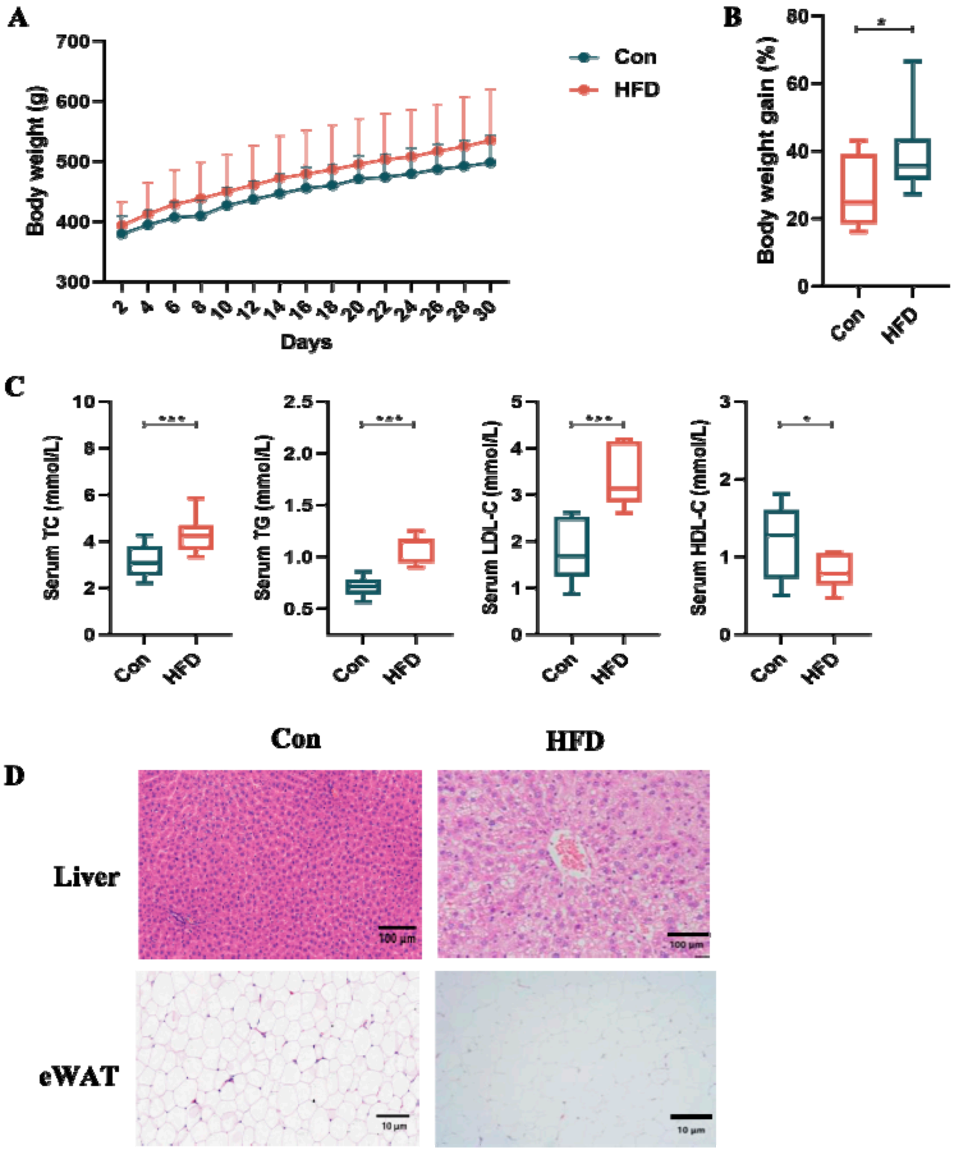

Supplementary Figure S1. The effect of high-fat diet intervention on SD rats. 20 SD rats were randomly divided into two groups, which were fed with a chow diet (1010086, n=10, Con group) and a high fat diet (XT19004, n=10, HFD group). These treatments lasted for 30 days. Compared with the Con group, HFD group could increase (A) Body weight, (B) Body weight gain, (C) Serum TC, TG, LDL-C and reduce HDL-C levels, (D) Histochemical staining showed that high-fat diet treatment led to hepatic steatosis and lipid accumulation in adipocytes. These results indicated that high-fat diet intervention could lead to lipid metabolism disorders in rats. Values are presented as mean ± SEM. **p*<0.05, ***p*<0.01 and ****p*<0.001 versus HFD group.

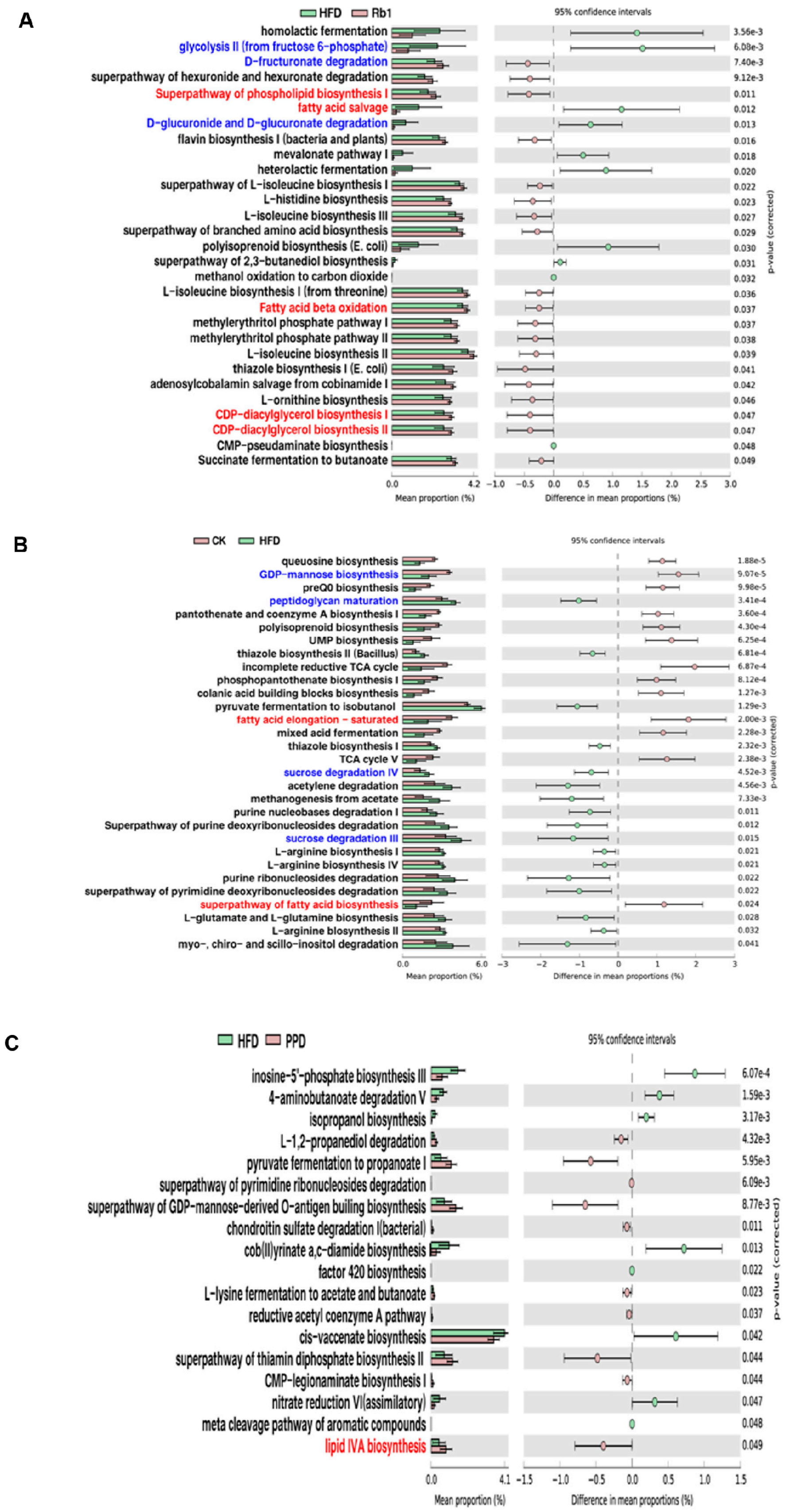

Figure S2. Function prediction in HFD-fed rats after ginsenoside Rb1, CK and PPD treatment. Prediction of the function of microbial genes involved in metabolism by PICRUSt analysis and based on the Welch's t-test ($p < 0.05$), the colored circles represent the 95% confidence intervals calculated by Welch's inverted method, (A) Rb1 vs. HFD, (B) CK vs. HFD, (C) PPD vs. HFD.
